# Functional Characterization of the Type III Secretion ATPase SsaN Encoded by *Salmonella* Pathogenicity Island 2

**DOI:** 10.1371/journal.pone.0094347

**Published:** 2014-04-10

**Authors:** Yukie Yoshida, Tsuyoshi Miki, Sayaka Ono, Takeshi Haneda, Masahiro Ito, Nobuhiko Okada

**Affiliations:** Department of Microbiology, School of Pharmacy, Kitasato University, Tokyo, Japan; Centre National de la Recherche Scientifique, Aix-Marseille Université, France

## Abstract

A type III secretion system (T3SS) is utilized by a large number of gram-negative bacteria to deliver effectors directly into the cytosol of eukaryotic host cells. One essential component of a T3SS is an ATPase that catalyzes the unfolding of proteins, which is followed by the translocation of effectors through an injectisome. Here we demonstrate a functional role of the ATPase SsaN, a component of *Salmonella* pathogenicity island 2 T3SS (T3SS-2) in *Salmonella enterica* serovar Typhimurium. SsaN hydrolyzed ATP *in vitro* and was essential for T3SS function and *Salmonella* virulence *in vivo*. Protein-protein interaction analyses revealed that SsaN interacted with SsaK and SsaQ to form the C ring complex. SsaN and its complex co-localized to the membrane fraction under T3SS-2 inducing conditions. In addition, SsaN bound to *Salmonella* pathogenicity island 2 (SPI-2) specific chaperones, including SsaE, SseA, SscA, and SscB that facilitated translocator/effector secretion. Using an *in vitro* chaperone release assay, we demonstrated that SsaN dissociated a chaperone-effector complex, SsaE and SseB, in an ATP-dependent manner. Effector release was dependent on a conserved arginine residue at position 192 of SsaN, and this was essential for its enzymatic activity. These results strongly suggest that the T3SS-2-associated ATPase SsaN contributes to T3SS-2 effector translocation efficiency.

## Introduction

A number of gram-negative pathogenic bacteria utilize a type III secretion system (T3SS) for their interactions with eukaryotic host cells. T3SS delivers bacterial effectors through a needle-like structure that extends across the inner and outer membranes of a bacterium and into the cytosol of eukaryotic cells [1], [2]. *Salmonella enterica* serovar Typhimurium (*S*. Typhimurium) is an enteropathogenic bacterium that causes gastroenteritis in humans and typhoid-like fever in mice. The virulence potential of *S*. Typhimurium is predominantly attributed to horizontally acquired genomic islands, termed as *Salmonella* pathogenicity island 1 (SPI-1) and SPI-2, which encode for different T3SSs. SPI-1 T3SS (T3SS-1) facilitates host cell invasion and inflammation [3], [4], whereas SPI-2 T3SS (T3SS-2) mediates intracellular survival and immune evasion [5], [6].

A functional T3SS requires five different types of proteins including chaperone, translocator, effector, apparatus protein, and transcriptional regulator. The structure of a T3S apparatus, known as an injectisome, is conserved among different pathogenic T3SSs and resembles flagellar T3SS [7], [8]. An injectisome consists of a structurally conserved basal body, which contains two pairs of rings that span the inner membrane and outer membrane, and is connected to a cytoplasmic C ring. Upon contact with a host cell during infection, the injectisome of a pathogenic bacterium extends its needle-like structure that protrudes outside the cell with a pore-forming protein (translocator) at the distal tip for delivery of effectors [9].

Recent studies have provided some evidence of the order in which a T3SS injectisome is assembled [10]–[13]. In *Yersinia*, the entire T3SS injectisome assembly process is initiated by the formation of an YscC secretin ring in the outer membrane, and then proceeds sequentially to form an inner ring composed of the single-pass inner membrane protein YscD and the inner membrane lipoproteins YscJ. After the assembly of these membrane rings is completed, an ATPase-C ring complex formed by YscK, YscL, YscN, and YscQ assembles at the cytosolic side of the injectisome where the ATPase forms a hexameric ring [14], which allows for the subsequent steps that result in translocator/effector secretion.

This ATPase activity coupled with proton motive force is important for providing energy for secretion [15], [16]. Several T3S-associated ATPases, including EscN from *E. coli* [17], YscN from *Yersinia* [18], [19], and InvC from *Salmonella* [20], have been characterized in terms of their activities and effector translocation functions via a T3SS. The T3S-associated ATPase protein family has been shown to have significant sequence homology to the β subunit of F_0_F_1_ ATPase and for hydrolyzing ATP. In addition, this ATPase is believed to play a role in the dissociation of a chaperone/effector complex before secretion and the subsequent unfolding of effectors [21].

SsaN is the ATPase associated with T3SS-2 and has significant sequence homology with other T3S-associated ATPases, such as InvC, YscN, and EscN [9], [22]. In this study, we enzymatically characterized SsaN as an ATPase associated with the T3SS of animal pathogenic bacteria. We demonstrated that SsaN is essential for secretion and *Salmonella* virulence. In addition, we found that SsaN interacted with the cytoplasmic SPI-2 component SsaK and the inner membrane protein SsaQ, which suggested that these proteins formed a C ring complex that assembled in a location adjacent to the inner bacterial membrane. *In vitro* assays revealed that SsaN dissociated a complex between the T3SS-2 specific chaperone SsaE and the effector/translocator protein SseB in an ATP-dependent manner.

## Materials and Methods

### Ethics statement

All animal experiments were approved by the Kitasato University Institutional Animal Care and Use Committee (Permit Number: J96-1) and were performed in accordance with the Regulations for the Care and Use of Laboratory Animals of Kitasato University and with the National Research Council Guide for the Care and Use of Laboratory Animals of Japan.

### Bacterial strains, plasmids, and growth conditions

The *Salmonella* strains and plasmids used in this study are listed in Table 1. *S.* Typhimurium strain SL1344 [23] was used as the wild-type strain, and isogenic deletion mutant strains were constructed using the lamda Red disruption system [24]. Double mutant strains were created by phage P22-mediated transduction. *Escherichia coli* DH5α (Takara Bio Inc.) was used for molecular cloning and the expression of recombinant proteins. *E. coli* strain S17.1 lamda *pir* was used for propagating π-dependent plasmids and for conjugation [25]. Bacteria were routinely grown overnight in LB broth (Sigma-Aldrich) at 37°C with aeration. To induce the expression of T3SS-2 genes, *Salmonella* strains were grown in low phosphate, low magnesium-containing medium (LPM) at pH 5.8 [26]. Ampicillin (100 μg/ml), chloramphenicol (25 μg/ml), kanamycin (25 μg/ml), and streptomycin (25 μg/ml) were used as required.

**Table 1:** *Salmonella* strains and plasmids used in this study.

Strains	Description	Source
SL1344	serovar Typhimurium, wild-type	[23]
TM4239	Nonpolar deletion of *ssaN* (Δ*ssaN*)	This study
TM1997	Nonpolar deletion of *ssaD* (Δ*ssaD*)	This study
TM203	Double mutant, Δ*ssaN*, Δ*ssaD*::*kan*	This study

Plasmids	Description	Source
pMW119	Low-copy-number expression vector	Nippon Gene
pFLAG-CTC	FLAG-fusion vector	NEB
p2HA-CTC	2HA fusion vector	[27]
pGEX-6P-1	GST fusion vector	GE Healthcare
pBAD-*Myc*-HisC	Myc-His6 fusion vector	Invitrogen
pACPJ-2HA	pACYC184 derivative containing 2HA with *sseJ* promoter	[27]
pFLAG-SsaN	pFLAG-CTC expressing SsaN-FLAG fusion protein	This study
pSsaN-FLAG	pMW119 expressing SsaN-FLAG fusion protein	This study
pSseJ-2HA	pACPJ-2HA expressing SseJ-2HA fusion protein	This study
pSsaK-2HA	p2HA expressing SsaK-2HA fusion protein	This study
pSsaQ-2HA	p2HA-CTC expressing SsaQ-2HA fusion protein	This study
pSsaE-2HA	p2HA-CTC expressing SsaE-2HA fusion protein	This study
pSseA-2HA	p2HA-CTC expressing SseA-2HA fusion protein	This study
pSscA-2HA	p2HA-CTC expressing SscA-2HA fusion protein	This study
pSscB-2HA	p2HA-CTC expressing SscB-2HA fusion protein	This study
pSsaN-2HA	p2HA-CTC expressing SsaN-2HA fusion protein	This study
pGEX-SsaE	pGEX-6p-1 expressing GST-SsaE	This study
pFLAG-SseB	pFLAG-CTC expressing SseB-FLAG fusion protein	This study
pBAD-SsaN-MH	pBAD-*Myc*-HisC expressing SsaN-Myc-His6 fusion protein	This study
pBAD-SsaN-R192G-MH	pBAD-*Myc*-HisC expressing SsaN-R192G-Myc-His6 fusion protein	This study
pBAD-EscN-MH	pBAD-*Myc*-HisC expressing EscN-Myc-His6 fusion protein	This study

### Plasmid construction

To construct plasmids that encoded for epitope-tagged fusion proteins, DNA fragments that contained the genes of interest were amplified by PCR with specific primers (Table 2), and cloned into pGEX-6P-1 (GE Healthcare) for N-terminal GST-tagged fusion proteins and pFLAG-CTC (Sigma-Aldrich), p2HA-CTC [27], and pBAD-*Myc*-HisC (Invitrogen) for C-terminal FLAG, 2HA, and Myc-His_6_-tagged fusion proteins, respectively.

**Table 2:** Primers used in this study.

Primer	Sequence (5′ to 3′)*
ssaN-red-FW	GATGCAACGTCTGAGGCTGAAATATCCGCCCCCCGATGGTGTGTAGGCTGGAGCTGCTTC
ssaN-red-RV	ATTGCTTTTCACGCCGCGCGATTATCTCCAGCAAAGTTTCCATATGAATATCCTCCTTAG
ssaD-red-FW	CTCAGTAGTAAATAATGGCATATCTCATGGTTAATCCAAAGTGTAGGCTGGAGCTGCTTC
ssaD-red-RV	CTAATGGATAGTTAATCAAAGTATCATAATGTTTAATCGTCATATGAATATCCTCCTTAG
SseJ-XhoI-FW	AAACTCGAGTTGAGTGTTGGACAGGGTTAT
SseJ-BamHI-RV	CCCGGATCCTTCAGTGGAATAATGATGAGC
SsaN-XhoI	GGGCTCGAGAATGAATTGATGCAACGTCTG (XhoI)
SsaN-BamHI	CCCGGATCCCTCGGTGAGTATTTGGTGTAA (BamHI)
FLAG-SphI-FW	CCTGCATGCTCACACAGGAGATATCATCTG (SphI)
FLAG-BamHI-RV	CCCGGATCCTATTGTCTCATGAGCGGATAC (BamHI)
SsaK-XhoI	GGCCTCGAGATGAGTTTTACTTCACTTCCT (XhoI)
SsaK-BglII	GGCAGATCTAAAAGAGGTAGCGATGAATAT (BglII)
SsaQ-XhoI	GGCTCGAGTTAAGAATAGCGAATGAAGAGC (XhoI)
SsaQ-BglII	GGAGATCTCGCTGTATTTTTGCAAAGATAC (BglII)
SscA-XhoI	GGACTCGAGAAAAAAGACCCGACCCTACAA (XhoI)
SscA-BglII	GGAAGATCTGCTCCTGTCAGAAAGTTGCTG (BglII)
SscB-XhoI	GGCCTCGAGATGATGAAAGAAGATCAGAAA (XhoI)
SscB-BglII	GGCAGATCTAGCAATAAGAGTATCAACCAT (BglII)
SsaE-gst-BamHI	ACCGGATCCACAACTTTGACCCGGTTAGAA (BamHI)
SsaE-gst-XhoI	AGGCTCGAGTTACTCTTGCTCACTCACTAC (XhoI)
SsaN-Myc-His-XhoI	GGCCTCGAGGAAGAATGAATTGATGCAACGTC (XhoI)
SsaN-Myc-His-KpnI	GGCGGTACCCTCGGTGAGTATTTGGTGTAATTT (KpnI)
SsaN-R192G-FW	GTTCTGGTGTTAATTGGTGAAGGTGGACGAGAAG
SsaN-R192G-RV	TTCACCAATTAACACCAGAACATTGCTGTC
EscN-Myc-His XhoI	GGCCTCGAGAATTTCAGAGCATGATTCTGTAT (XhoI)
EscN-Myc-His KpnI	GGTGGTACCGGCAACCACTTTGAATAGGCTTT (KpnI)

*Letters in bold indicate restriction site shown in parenthesis.

To construct the complementing pSsaN plasmid that expressed SsaN-FLAG fusion proteins, the *ssaN-flag* gene was amplified from the pFLAG-SsaN plasmid using the primers FLAG-SphI-FW and FLAG-BamHI-RV (Table 2), and then ligated into a low-copy-number pMW119 vector (Nippon Gene).

A point mutation in the *ssaN* gene was created with a QuikChange Site-directed mutagenesis kit (Stratagene) using the primers SsaN-R192G-FW and SsaN-R192G-RV (Table 2) to replace arginine with glycine at position 192 in SsaN. This mutation was confirmed by DNA sequencing.

### Antibodies

Rabbit polyclonal anti-*Salmonella* LPS O4 antibody (Denka Seiken) was used at a dilution of 1∶5,000. Mouse polyclonal anti-SseB [28] and rabbit polyclonal anti-Omp proteins [29] antibodies were used as described. Mouse monoclonal antibodies anti-FLAG (1∶20,000) (Sigma-Aldrich), anti-GST (1∶2,000) (Upstate), and anti-DnaK (1∶2,000) (Calbiochem) were used as primary antibodies for immunoblotting. A mouse monoclonal anti-HA epitope tag HA.11 was used at a dilution of 1∶2,000 (Covance) for immunofluorescence microscopy and immunoblot analysis. Alexa488-conjugated goat anti-mouse IgG and Alexa594-conjugated goat anti-rabbit IgG secondary antibodies (dilutions of 1∶500) were obtained from Invitrogen. Alkaline phosphatase-conjugated goat anti-mouse IgG and anti-rabbit IgG antibodies (Sigma-Aldrich) were used at dilutions of 1∶20,000.

### Cell culture, bacterial infection, and immunofluorescence microscopy

HeLa cells were grown in Eagle's minimal essential medium (MEM; Sigma-Aldrich) supplemented with 10% FBS and gentamicin (100 μg/mL) and kanamycin (60 μg/mL) at 37°C in a 5% CO_2_ atmosphere. Bacterial infection of HeLa cells was done as previously described [28]. For immunofluorescence straining, cells were fixed with 4% paraformaldehyde permeabilized with 0.1% TritonX-100, and probed with different antibodies as described [28]. Labeled cells were analyzed with a Zeiss confocal laser scanning microscope (LSM510 META).

### ATPase assay

SsaN-Myc-His_6_, SsaN_R192G_-Myc-His_6_, and EscN-Myc-His_6_ fusion proteins were overexpressed in *E. coli* TOP10 (Invitogen) strains and purified using Ni-NTA agarose (Qiagen). The ATPase activities of these proteins were determined using a malachite green assay (BIOMOL GREEN; Enzo Life Sciences International, Inc). For all experiments, specific activity was determined using the equation for a standard curve generated using phosphate standards. Reaction mixtures contained varying amounts of purified proteins, 4 mM ATP, 100 mM Tris-HCl (pH 8.0), and 4 mM MgCl_2_. The reaction mixture (1 ml) was first incubated at 37°C for 30 min and then at room temperature for 30 min. The absorbance of triplicate samples was measured at 620 nm. To calculate the amounts of released phosphate, test sample absorbance was compared with the absorbance of a phosphate standard curve according to the manufacturer's instructions.

### FLAG pull-down assay

FLAG fusion protein complexes were immunoprecipitated from *E. coli* lysates using FLAG beads conjugated with an anti-FLAG M2 antibody (Sigma-Aldrich) as previously described [27]. Briefly, Bacterial lysate containing FLAG fusion protein was incubated with FLAG beads at 4°C for 2 h. Beads were washed five times with ice-cold Tris-buffered saline (TBS) at pH 7.6 and lysates from *E. coli* containing 2HA fusion proteins were further incubated with FLAG fusion protein immobilized-beads. After a final washing, bound proteins were competitively eluted from the beads using FLAG peptides (Sigma-Aldrich) at a final concentration of 100 μg/ml. The eluted protein fraction was then analyzed by SDS-PAGE and Western blotting.

### Subcellular fractionation

*Salmonella* strains were grown in 20 ml of LPM medium (pH 5.8) at 37°C for 16 h and then used for cytoplasmic and membrane fraction isolation according to the method of Gauthier *et al.* [30]. The culture supernatant fraction was obtained as previously described [26]. Samples were run on 12% SDS-PAGE and transferred to PVDF membranes (Immobilon, Millipore). The membranes were hybridized with different antibodies and developed using a Sigma Fast BCIP/NBT detection system as previously described [28].

### Chaperone release assay

For a chaperone release assay, GST-SsaE was expressed in *E. coli*, immobilized on glutathione-Sepharose (GE Healthcare) as previously described [27], and incubated at 4°C for 2 h with an *E. coli* lysate that contained SseB-FLAG. The Sepharose matrix was washed five times with TBS (pH 7.6) to remove unbound proteins and incubated with 1 μg of purified SsaN-Myc-His_6_ or SsaN_R192G-Myc-His6_ in the presence of 150 μM ATP or ATPγS for 1 h. Unbound proteins were removed by centrifugation, the supernatant was precipitated with trichloroacetic acid, and then resuspended in 20 μl of Laemmli buffer. Bound proteins (10 μl) and 10 μl of precipitated supernatants were analyzed by SDS-PAGE and immunoblotting.

### Mouse infection

Female BALB/c mice (5- to 6-weeks old) were used for the mixed infection assays. Three to five mice were inoculated intraperitoneally with a mixture of two strains comprising 5×10^4^ cfu of each strain in physiological saline. The numbers of viable bacteria in infected spleens were determined at 48 h after infection, as previously described [28]. Each competitive index (CI) value was the mean of at least three independent infections ± SD. CI results were compared by Student's *t* test.

## Results

### SsaN is crucial for SPI-2 effector protein translocation

**Figure 1:**
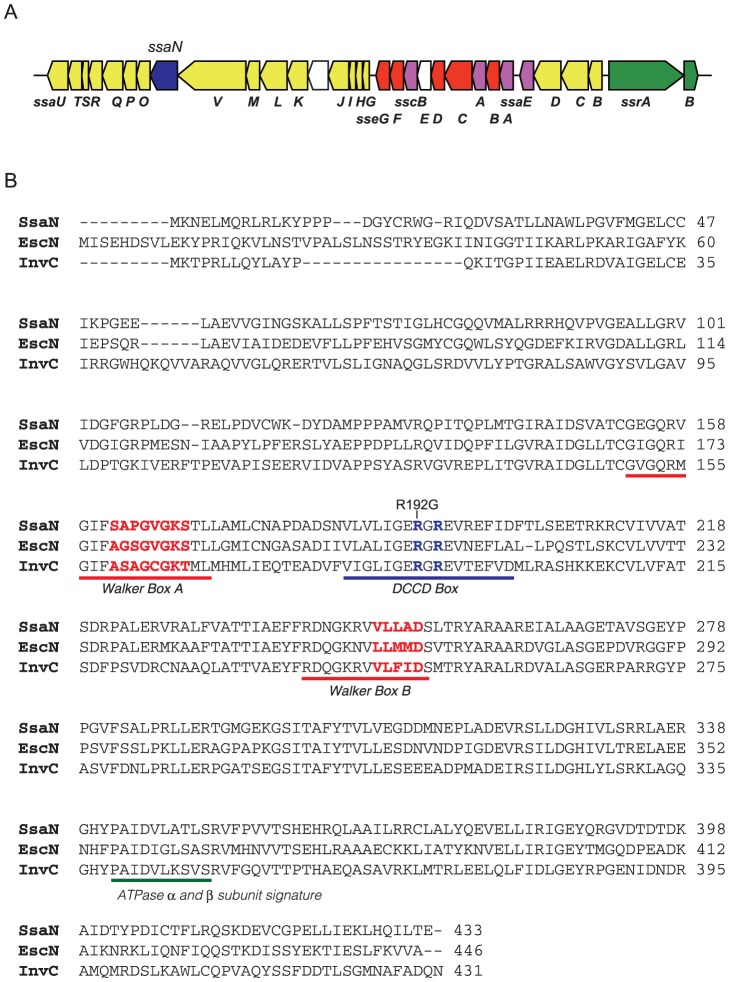
*ssaN* encodes for a protein with the conserved sequence of other T3SS ATPases. (**A**) Genetic organization of the T3SS-2 genes involved in *S*. Typhimurium virulence. Genes encoding for proteins for apparatus (*yellow*), effectors and translocators (*red*), chaperones (*purple*), and regulators (*green*) are shown. The *ssaN* gene is indicated by *blue*. The genes *ssaDCB* and *ssrA* are also referred to as *spiBAC* and *spiR*, respectively. (**B**) Amino acid sequence comparisons of SsaN with the EPEC T3SS ATPase EscN and *Salmonella* T3SS-1 ATPase InvC. The locations of known predicted motifs within the amino acid sequence of SsaN, including Walker Boxes A and B, the dicyclohexylcarbodiimide-binding site (DCCD) box, and the ATPase α and β subunit signature sites, are indicated.

In the T3SS-2 locus of *S*. Typhimurium, *ssaN* is flanked by *ssaO* and *ssaV* (**Fig. 1A**). SsaN is 433 amino acids in length and has a predicted molecular mass of 47.8 kDa. SsaN shares significant sequence homology with other T3S-associated ATPases, including InvC from *Salmonella* SPI-1, EscN from *E. coli*, and YscN from *Yersinia* (**Table 3**). The C-terminal regions (catalytic domains) of these proteins contain conserved amino acid residues that are characteristic of Walker box A and B motifs of P-loop nucleoside triphosphate hydrolases, which are also found in SsaN (**Fig. 1B**).

**Table 3:** Nomenclature of flagellar and pathogenic T3SS components.

SPI-2 Protein	Putative function	Flagellar T3SS	*S*. Typhimurium SPI-1	Yersinia	Shigella	EPEC
SsaN	ATPase	FliI	InvC	YscN	Spa47	EscN
SsaK	ATPase complex	FliH	OrgB	YscL	MxiN	EscL
SsaQ	C-ring	FliN + FliM	SpaO	YscQ	Spa33	EscQ
SsaD	Basal body	FliG	PrgH	YscD	MxiG	EscD

**Figure 2:**
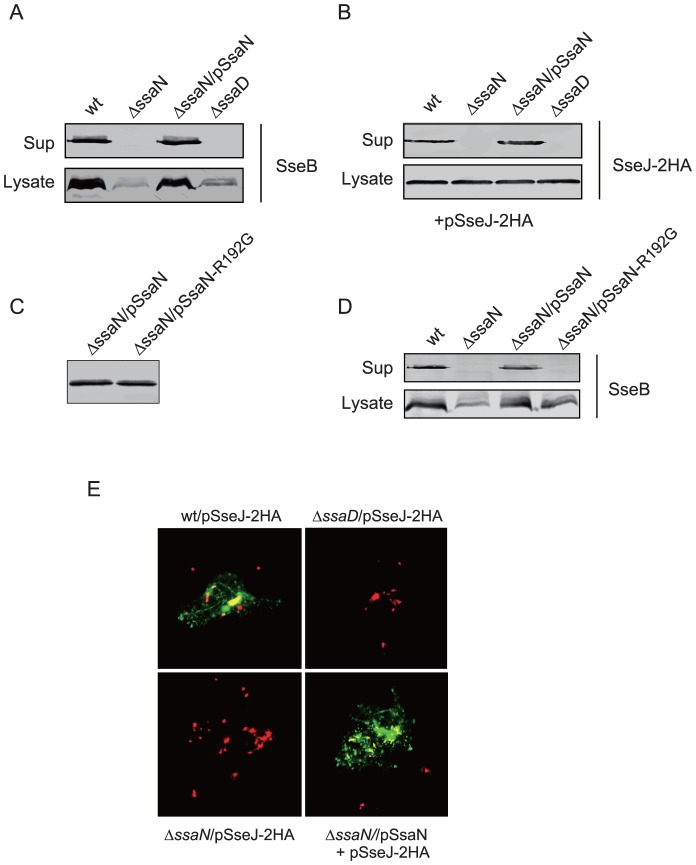
SsaN is required for *Salmonella* T3SS-2 dependent secretion. (**A**) *Salmonella* wild-type, *ΔssaN* mutant, *ΔssaN* mutant complemented with cloned *ssaN*, and *ssaD* mutant strains were grown in LPM medium (pH 5.8). Protein samples from equal numbers of bacteria were analyzed by Western blotting using an anti-SseB antibody for the secreted protein fraction (Sup) and whole cell lysates (Lysate). (**B**) *Salmonella* wild-type and mutant strains that harbored pSseJ-2HA plasmid were grown in LPM medium (pH 5.8). Protein samples from equal numbers of bacteria were subjected to Western blot analysis to detect SeeJ-2HA and SseB in secreted protein fractions (Sup) and in whole cell lysates (Lysate). (**C**) *Salmonella* wild-type, *ΔssaN* mutant, *ΔssaN* mutant complemented with cloned *ssaN*, and *ΔssaN* mutant complemented with point mutated *ssaN_R192G_* strains were grown in LPM medium (pH 5.8). Protein samples from equal numbers of bacteria were analyzed by Western blotting with an anti-SseB antibody for the secreted protein fraction (Sup) and whole cell lysates (Lysate). (**D**) Confocal immunofluorescence analysis of HeLa cells infected with *Salmonella* strains that expressed SseJ-2HA. Infected cells were fixed at 8 h after infection. Bacteria were labeled with a rabbit anti-*Salmonella* LPS O4 antibody (*red*), and a mouse anti-HA antibody was used to detect HA-fusion proteins (*green*). All these experiments were repeated independently three times with similar results. Representative images are shown.

To investigate the function of SsaN in T3SS-2, an *ssaN* deletion mutant was constructed, and then we examined the ability of this *ssaN* mutant strain to secrete the translocator protein SseB into a culture supernatant. When *Salmonella* strains were grown in LPM medium at pH 5.8 under conditions that induced T3SS-2 expression, SseB secretion from the *ssaN* mutant strain was undetectable, similar to an *ssaD* (gene predicted to encode for a component of the T3SS-2 apparatus (**Table 3**) and also referred to as *spiB*) mutant strain (**Fig. 2A**). Consistent with previous results [31], we found reduced accumulation of cytosolic SseB in *Salmonella* T3S apparatus deficient mutants.

Therefore, we next examined whether SsaN was required for the export of another T3SS-2 effector, SseJ, which is encoded outside of the T3SS-2 region. SseJ (SseJ-2HA) in whole cell lysates of *ssaD* and *ssaN* mutant strains that expressed SseJ-2HA from a plasmid were detected at the same level as those of the wild-type strain, whereas a loss of T3SS-2 function completely prevented SseJ secretion into a culture supernatant (**Fig. 2B**). This secretion deficiency by the *ssaN* mutant was complemented by introducing a plasmid that expressed FLAG-tagged SsaN (SsaN-FLAG). These results strongly suggested that effector secretion depended on the presence of SsaN.

To further investigate whether SsaN function depended on its ATPase activity, the conserved arginine residue at position 192 of SsaN located in the dicyclohexylcarbodiimide-binding site (DCCD box) in its catalytic domain (**Fig. 1B**), which is essential for ATPase activity [20], was replaced by glycine. The point-mutated derivative SsaN_R192G_-FLAG fusion protein was stable because both SsaN-FLAG and SsaN_R192G_-FLAG were detectable in comparable amounts in whole cell extracts (**Fig. 2C**). As expected, introducing a plasmid that expressed SsaN_R192G_ into the Δ*ssa* *N* mutant failed to complement SseJ secretion (**Fig. 2D**).

We also examined the ability of the Δ*ssa* *N* mutant strain to translocate the T3SS-2 effector protein SseJ into HeLa cells by confocal immunofluorescence microscopy. SseJ-2HA was translocated across the vacuolar membranes of HeLa cells that were infected with the wild-type strain. SseJ was also translocated by the complemented *ssaN* mutant strain, whereas SseJ-2HA translocation was not detected in cells that were infected with the *ssa* *N* mutant strain (**Fig. 2E**).

### SsaN ATPase activity *in vitro*

**Figure 3:**
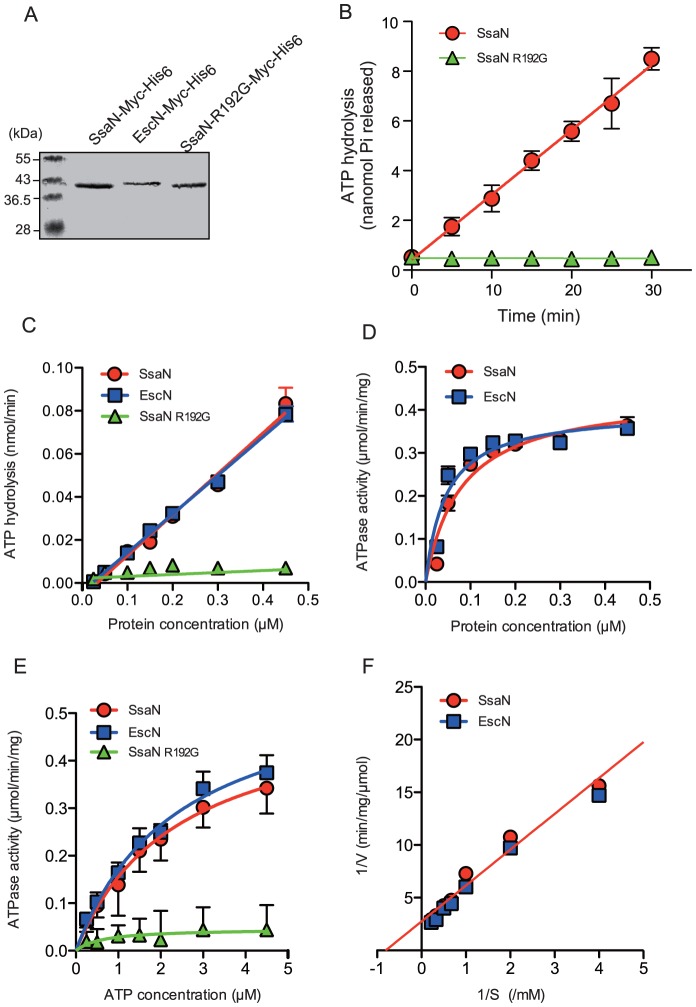
SsaN ATPase activity. (**A**) SsaN-Myc-His_6_, EscN-Myc-His_6_, and SsaN_R192G_-Myc-His_6_ were purified by nickel affinity chromatography after elution with 500 mM imidazole. Proteins were analyzed by SDS–PAGE and stained with Coomassie brilliant blue. (**B**) ATP hydrolysis by purified SsaN-Myc-His_6_ and SsaN_R192G_-Myc-His_6_ fusion proteins was determined using a malachite green assay with 4 mM ATP in the presence of 4 mM MgCl_2_ over a period of 30 min after adding the malachite green reagent. Error bars indicate standard deviations. (**C**) ATP hydrolysis with different concentration of SsaN-Myc-His_6_, EscN-Myc-His_6_, and SsaN_R192G_-Myc-His_6_ fusion proteins. Error bars indicate standard deviations. (**D**) ATPase activity with different concentration of SsaN-Myc-His_6_ and EscN-Myc-His_6_. Error bars indicate standard deviations. (**E**) ATPase activity of SsaN-Myc-His_6_, EscN-Myc-His_6_, and SsaN_R192G_-Myc-His_6_ with ATP concentrations ranging from 1 to 5 mM. Error bars indicate standard deviations. (**F**) Data acquired in panel E were used to generate a Lineweaver–Burk plot. S: substrate, V: velocity of the enzymatic reaction.

To test whether SsaN could hydrolyze ATP *in vitro*, SsaN-Myc-His_6_, SsaN_R192G_-Myc-His_6_, and EscN-Myc-His_6_ were overexpressed in *E. coli* cells and then purified using Ni-NTA agarose (**Fig. 3A**). Purified fusion proteins were subjected to a colorimetric malachite green assay to measure phosphate release. Purified SsaN-Myc-His_6_ hydrolyzed ATP in a linear, time-dependent manner with a mean ATPase activity of 0.36±0.06 μmol/min/mg, whereas a purified SsaN_R192G_-Myc-His_6_ fusion protein did not show any significant ATPase activity (**Fig. 3B**).

We also determined SsaN specific activity at different protein concentrations. These results showed a non-linear increase in ATPase activity (**Fig. 3C**), which indicated positive cooperativity of SsaN similar to that reported for other T3S-associated ATPases [17], [18], [32]. SsaN enzymatic activity assays at different substrate concentrations showed an increase in ATP activity with 5 mM ATP (**Fig. 3D**). The *Km* value for ATP was 0.81±0.02 mM similar to the *Km* value of EscN (0.73±0.01 mM), as determined using a Lineweaver–Burk plot (**Fig. 3E**).

### SsaN interacts with C ring complex and chaperone proteins

It has been shown that T3S-associated ATPases interacted with components of a C ring complex, the most proximal part of the basal body of type III apparatus [33]-[37]. In the *Salmonella* flagellar T3SS, this complex consists of FliI, FliH, and FliN [37] and that of *Yersinia* T3SS consists of YscN (FliI homolog), YscL (FliH homolog), YscK, and YscQ (FliN homolog) [33] (**Table 3**). Thus, to investigate whether SsaN interacted with conserved C ring components of this secretion apparatus, we tested for possible interactions between SsaN, SsaK (YscL/FliH homolog), and SsaQ (YscQ/FliN homolog) (**Table 3**). To date, to the best of our knowledge, no homolog of YscK has been identified in *Salmonella* T3SS-2.

**Figure 4:**
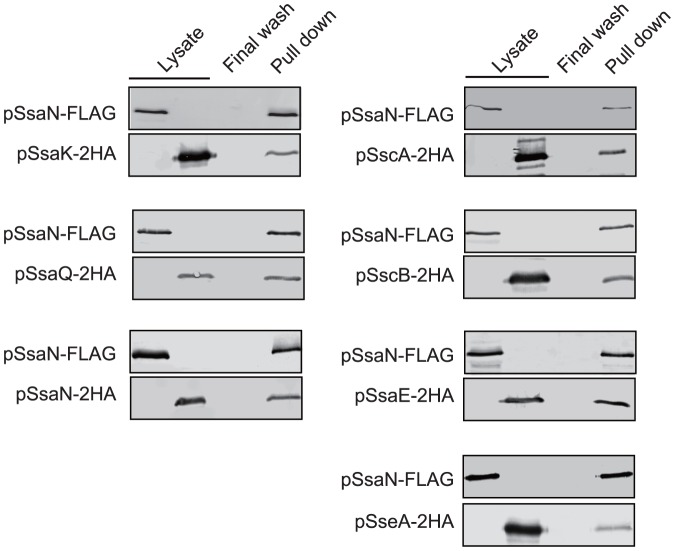
SsaN interactions with the T3SS-2 C-ring components SsaK and SsaQ (A) and T3SS-2 specific chaperones (B). SsaN interactions with different proteins were analyzed using FLAG pull-down assays. SsaN-FLAG was expressed in *E. coli* strains. SsaN-FLAG fusion proteins in lysates were immobilized using anti-FLAG affinity beads. Bacterial lysates from *E. coli* strains that expressed SsaK-2HA, SsaQ-2HA, SsaN-2HA, SscA-2HA, SscB-2HA, SsaE-2HA, and SseA-2HA were then incubated with SsaN-FLAG immobilized-beads. After a final washing with beads (Final wash), protein-binding affinity beads were eluted by adding FLAG peptides. Lysates and eluted proteins (Elute) were detected with anti-FLAG and anti-HA antibodies.

To examine the interactions of SsaN with SsaK and SsaQ, pull-down assays were used with SsaN-FLAG fusion proteins that were expressed in *E. coli* strains. Cell lysates that contained SsaK-2HA and SsaQ-2HA fusion proteins were incubated with anti-FLAG beads immobilized with SsaN-FLAG. Bound proteins were analyzed by SDS-PAGE and immunoblotting using an anti-HA antibody. These results showed that SsaN co-eluted with SsaK-2HA and SsaQ-2HA (**Fig. 4A**). Similar results were obtained using an enzymatically inactive SsaN_R192G_-FLAG derivative (data not shown).

As it has been thought that T3S-associated ATPase binds chaperone molecule that is associated with translocator/effector protein, followed by dissociation of this complex. Therefore, we next examined for interactions between SsaN and the T3SS-2 specific chaperones SsaE, SseA, SscA, and SscB. Cell lysates from *E. coli* strains that expressed SsaE-2HA, SseA-2HA, SscA-2HA, and SscB-2HA were incubated with anti-FLAG beads conjugated with SsaN-FLAG. Bound proteins were detected with an anti-HA antibody. As shown in Figure 4B, SsaN bound to all of the T3SS-2 chaperones tested.

### Subcellular localizations of SsaN, SsaK, and SsaQ

**Figure 5:**
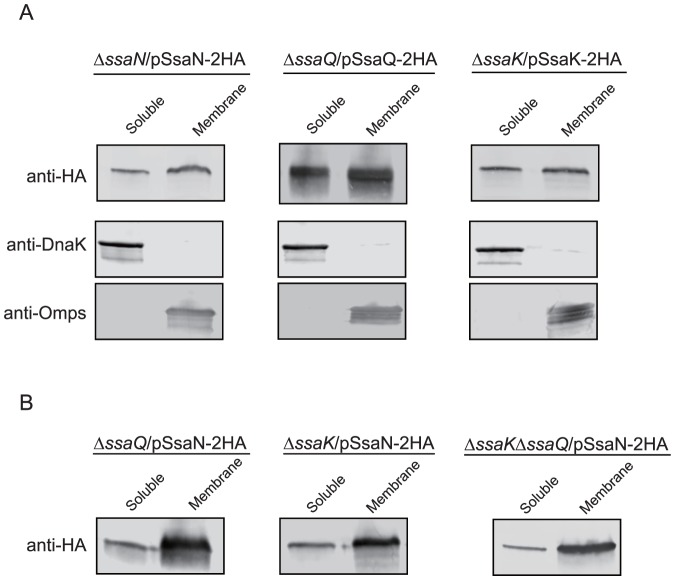
Subcellular localizations of SsaN, SsaK, and SsaQ. SsaN, SsaK, and SsaQ co-localized to bacterial membranes after T3SS activation. *Salmonella* strains that expressed 2HA-tagged SsaN, SsaK, or SsaQ were grown in LPM (pH 5.8) for 8 h under permissive conditions that induced T3SS-2 secretion, after which bacteria were fractionated into soluble (S) and membrane (M) fractions. These fractions were analyzed by immunoblotting using an antibody against 2HA epitopes. DnaK and outer membrane proteins (Omps) were used as markers for the cytoplasmic and membrane fractions, respectively. (**A**) Localization was examined in the complemented strains. (**B**) Localization of SsaN was examined in *ssaQ*, *ssaK*, and *ssaKssaQ* double mutant strains.

The interactions between SsaN, SsaK, and SsaQ suggested similar localizations for these proteins in the T3SS-2 injectisome. Thus, we examined their subcellular localizations by a bacterial cell fractionation method, as previously described [27], [30]. *S.* Typhimurium *ssaN*, *ssaK*, and *ssaQ* mutant strains that expressed SsaN, SsaK, and SsaQ tagged with C-terminal 2-HA were grown in LPM medium (pH 5.8). Bacterial cells were then separated into soluble (cytoplasm) and membrane fractions. SsaN (SsaN-2HA), SsaK (SsaK-2HA), and SsaQ (SsaQ-2HA) were detected in both the soluble and membrane fractions (**Fig. 5A**). To determine whether the localization of SsaN in the membrane fraction required its binding to SsaK and/or SsaQ, cell fractionation was performed with *Salmonella ssaK* and *ssaQ* deletion mutant strains that expressed SsaN-2HA plasmids. SsaN localization was not affected by the absence of SsaK and SsaQ compared with the wild-type strain (**Fig. 5B**). These results indicated that SsaN could associate with the membrane regardless of the presence of the other ATPase-associated components.

### SsaN dissociates a complex between SseB and SsaE

**Figure 6:**
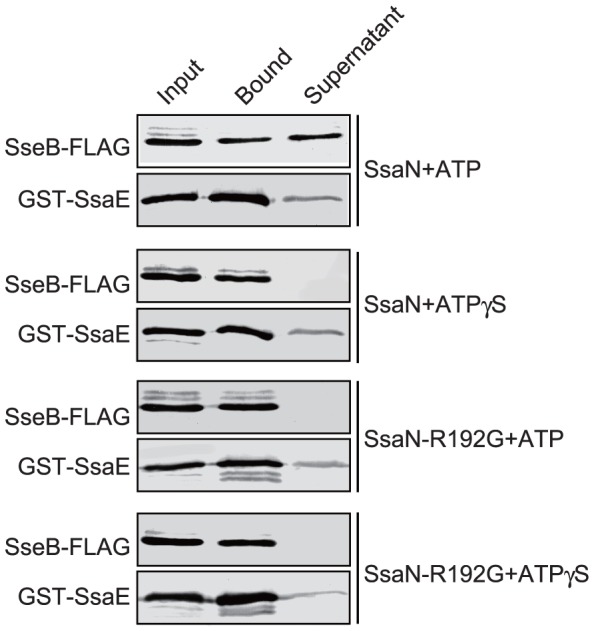
SsaN causes the release of the SsaE-bound effector/translocator protein SseB. Purified SsaN-Myc-His_6_ or SsaN_R192G_-Myc-His_6_ was incubated with GST-SsaE and SseB-FLAG protein complexes in the presence of ATP or the non-hydrolyzable ATP derivative ATP-γS, as indicated. Total cell lysates of *E. coli* that expressed SseB-FLAG, bound proteins, and proteins in the supernatant were analyzed by immunoblotting using anti-FLAG and anti-GST antibodies.

It was previously reported that the T3S-associated ATPase InvC from *S.* Typhimurium dissociated a chaperone-translocator/effector complex in an ATP-dependent manner [21]. To analyze if SsaN could dissociate some T3SS substrates from their cytoplasmic chaperones, we immobilized a complex consisting of a GST-SsaE fusion protein and the translocator protein SseB-FLAG on glutathione-Sepharose. SsaE has been shown to be a chaperone specific for SseB [27]. GST-SsaE/SseB-FLAG complex was incubated with purified SsaN-Myc-His_6_ in the presence of ATP [21] and proteins that were bound to and then released from the matrix were analyzed by immunoblotting using antibodies against GST and the FLAG epitope, respectively. As expected, GST-SsaE was found only in the matrix-bound fraction, whereas SseB-FLAG was also detected in the supernatant, which suggested that it was released from the chaperone (**Fig. 6**). We did not detect any SseB-FLAG release in the presence of the non-hydrolyzable ATP derivative ATPγS or the catalytically inactive SsaN_R192G_-Myc-His_6_ fusion protein (**Fig. 6**). The latter finding cannot be attributed to the lack of protein–protein interaction because SsaN_R192G_-Myc-His_6_ bound to SsaE (**Fig. 6**). In summary, our results indicate that SsaN releases the translocator protein SseB from the T3SS-2 specific chaperone SsaE in an ATP-dependent manner.

### SsaN is essential for *Salmonella* virulence

**Figure 7:**
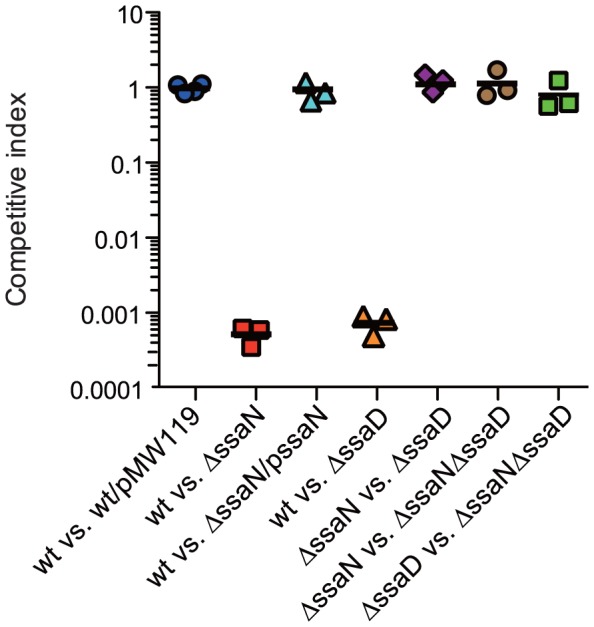
SsaN is required for T3SS-2 dependent mouse virulence *in vivo*. Mice were infected peritoneally with an equal mixture of the two indicated bacterial strains. The CI values for the spleen were determined at 48

To examine the virulence function of SsaN *in vivo*, we performed mixed infections in BALB/c mice. Control experiments with the wild-type strain and the wild-type strain that harbored the pMW119 plasmid resulted in CI of 1.13±0.19. The CI value of the wild-type strain versus the *ssaN* mutant strain was significantly reduced to 0.047±0.013 (*P*<0.01), compared to that of the *ssaD* mutant. In addition, the CI value of the wild-type strain versus the complemented-*ssaN* mutant that expressed an SsaN-FLAG fusion protein from a plasmid was 1.16±0.21, which was not significantly different from the CI value of the wild-type strain. This showed that the replication defect of the *ssaN* deletion mutant was due to a loss of SsaN function (**Fig. 7**). These results clearly demonstrated that *ssaN* contributed to *Salmonella* virulence in the mouse model of systemic infection.

Furthermore, to examine the interaction between SsaN and T3SS-2 *in vivo*, an *ssaN* mutation was introduced into the *ssaD* mutant strain and single and double mutant strains were analyzed by a competitive index as COI (canceled out competitive index) for mixed infections in mice. These results showed that the COI of a *Salmonella* strain that carried both *ssaN* and *ssaD* mutations compared to that of a single *ssaN* mutant strain was not significantly different from CI of the *ssaN* mutant strain compared with that of the *ssaD* mutant strain. Similarly, an *ssaNssaD* double mutant strain was no more attenuated than an *ssaD* single mutant (**Fig. 7**). Thus, SsaN appeared to contribute to *S.* Typhimurium virulence via T3SS-2 function during systemic infection.

## Discussion

The roles of T3S-associated ATPases during secretion and translocation of effectors have been studied both for animal and plant pathogenic bacteria. In *Salmonella*, the ATPase associated with T3SS-1, InvC, has been functionally studied [21], [38]. However, little is known regarding T3S-associated ATPases from T3SS-2. Here, we biochemically characterized SsaN as a T3S-associated ATPase for T3SS-2. Consistent with a previous study, SsaN had ATPase activity as determined by a pyruvate kinase–lactate dehydrogenase coupled assay [22], we found that SsaN hydrolyzed ATP in a linear, time-dependent manner with a *K_m_* of 0.81±0.02 mM and a *V_max_* of 0.36±0.06 μmol/min/mg. These results were comparable to those for other T3S-associated ATPases, including InvC [38], EscN [17], YsaN [39], HrcN [32], CdsN [40], and FliI [41], [42]. Furthermore, we demonstrated that the ATPase activity of SsaN was essential for secretion and *Salmonella* virulence.

Protein-protein interaction assays showed that SsaN interacted with other T3S components, including a cytoplasmic T3S component, SsaK, and a T3S structural inner membrane component, SsaQ. Similar protein organizations involving SsaN, SsaK, and SsaQ were found in the flagellar system and the T3SS in *Yersinia* and EPEC, in which FliI/YscN/EscN (SsaN homolog) was shown to bind to both FliH/YscL/EscL (SsaK homolog) and FliN/YscQ/EscQ (SsaQ homolog) [18], [33], [43]–[45]. FliH and YscL have been shown to negatively control the ATPase activities of FliI and YscN, respectively [43], [44]. In addition, HrcL (YscL homolog in *Xanthomonas campestris* pv. vesicatoria) positively regulates HrcN stability (YscN homolog). Thus, the sequence homology between SsaK and YscL/FliH suggests that it plays a similar role. However, the precise role of SsaK in T3SS-2 function has not been determined.

SsaQ belongs to the FliN/YscQ/EscQ family of flagellar and pathogenic T3SS proteins [46]. These proteins are the conserved core proteins of these structures and constitute a cytoplasmic platform (C ring) connected to the basal body of the T3SS injectisome. It was previously shown that the C ring proteins interacted with components of the secretion apparatus, including the ATPase and its regulatory proteins [33], [35]. These are required to form the needle-like structure of a T3SS [35] and for the assembly of the associated protein complex between an ATPase and the C ring [13]. The localizations of SsaN, SsaK, and SsaQ indicated that these proteins are found in both the cytoplasm and membrane. Therefore, it is most likely that these T3SS-2 proteins are part of the C ring complex that is linked to the inner membrane. Moreover, data from this and other studies [47], [48] demonstrated that *Salmonella* strains that carried a mutation in the *ssaN*, *ssaK*, or *ssaQ* gene abolished T3SS-2 function and became less virulent, which suggests that they are a part of the essential secretion apparatus of *Salmonella* T3SS-2.

We also found interactions between SsaN and T3SS-2 specific chaperones, including SseA, SsaE, SscA, and SscB. This also indicated that SsaN is the T3SS-2 specific ATPase of *S*. Typhimurium. Using an *in vitro* chaperone release assay [21], we showed that SsaN dissociated a complex between the translocator SseB and its specific chaperone molecule SsaE in an ATP-dependent manner. SsaN-mediated release of SseB was dependent on a conserved arginine residue in SsaN that was crucial for ATP hydrolysis and secretion. This indicates that the ATPase activity and, presumably, the dissociation of SsaE-effector complexes play a key role in T3SS-2 in *Salmonella*. Similar findings were reported for the T3S-associated ATPase InvC from *S*. Typhimurium [21] and HrcN from *X. campestris* pv. vesicatoria [32], which suggests that a T3S-associated ATPase is required for the release of chaperone-bound translocator/effector proteins. In addition, it was previously shown that SsaN interacted with SrcA, a chaperone for the T3SS-2 effectors SseL and PipB2 [22]. Therefore, the T3SS-2 specific ATPase SsaN may provide a binding site for chaperones and promote the secretion of chaperone-bound translocator/effector proteins via the T3SS-2 injectisome.

Recent work has shown alternative scenario regarding the docking of chaperone-substrate and ATPase. The type III chaperones CesAB and FlgN are shown to be unable to interact with cognate ATPase EscN and FliI, respectively, in the absence of the substrate effectors [49], [50]. Interaction of chaperone with its substrate is believed to cause a conformational switch that confers the binding competency to ATPase on the chaperone-substrate complex. Contrary, we here have shown that all type III chaperones tested in this study (SsaE, SseA, SscA and SscB) are able to directly interact with the ATPase, SsaN. Furthermore, almost all known type III chaperones were shown to bind to the cognate ATPase, such as CesT of EPEC [51], Spa13 of *Shigella flexneri* [52], HpaB of *X. campestris* pv. vesicatoria [32], and FliT of *S*. Typhimurium [53]. Much further work will be required to elucidate the type III ATPase function as a docking platform for chaperone-effector complexes and to explain the discrepancies of chaperone recognition by the type III-associated ATPase.

In general, the protein interaction networks within each T3SS are consistent with the weak similarities detected between components of the various T3SSs. Although YscL and YscQ protein families are commonly associated with ATPases in flagellar and pathogenic T3SSs, specific interactions between T3SS-associated ATPases with other T3SS components have been found in some bacteria. In addition to YscL and YscQ homologs, there is evidence for specific interactions between YscN and YscK in *Yersinia* [33], Spa47 and MxiK in *Shigella* [36], CdsN and CdsD in *Chlamydophila pneumoniae* [54], and HrcN and HrcU in *X. campestris* pv. vesicatoria [32]. Although T3SS component proteins homologous to YscK and MxiK proteins are absent in *Salmonella* T3SS-2, CdsD and HrcU proteins show weak sequence homology to inner membrane component proteins of the T3SS-2 injectisome, SsaD and SsaU, respectively. Thus, it will be important to determine whether SsaN interacts with these proteins with regard to SsaN function in T3SS-2.

Based on earlier studies, requirement of the specific ATPase in functional T3SS is common and general notion. In contrast, identification of the conserved arginine residue of T3SS ATPase crucial for its T3SS-associated function, namely effector secretion, ATPase activity and chaperone release, is a novel finding. At present, the precise role of SsaN in the assembly of the T3SS-2 remains mainly unknown. However, comparative analysis using the arginine mutant of SsaN will allow us to address this issue in future work.
